# MicroRNAs in Postischemic Vascular Repair

**DOI:** 10.1155/2012/486702

**Published:** 2012-03-15

**Authors:** Andrea Caporali, Costanza Emanueli

**Affiliations:** Laboratory of Vascular Pathology and Regeneration, Regenerative Medicine Section, School of Clinical Sciences, Bristol Heart Institute, University of Bristol, Bristol, BS2 8HW, UK

## Abstract

The term *angiogenesis* describes the growth of endothelial sprouts from preexisting postcapillary venules. More recently, this term has been used to generally indicate the growth and remodeling process of the primitive vascular network into a complex network during development. In adulthood, angiogenesis is activated as a reparative process during wound healing and following ischemia, and it plays a key role in tumor growth and metastasis as well as in inflammatory diseases and diabetic retinopathy. MicroRNAs (miRNAs) are endogenous, small, noncoding RNAs that negatively control gene expression of target mRNAs. In this paper, we aim at describing the role of miRNAs in postischemic angiogenesis. First, we will describe the regulation and the expression of miRNAs in endothelial cells. Then, we will analyze the role of miRNAs in postischemic vascular repair. Finally, we will discuss the role of circulating miRNAs as potential biomarkers in ischemic diseases.

## 1. Introduction

Postnatal neovascularization occurs in pathological diseases, including diabetic retinopathy, arthritis, and tumor growth and metastasis, as well as during wound healing and postischemic repair [1]. Following occlusion of a major artery, two different types of vascular regrowth responses are activated to contrast the ischemic condition and salvage of injured ischaemic tissue: sprouting of capillaries (angiogenesis) and growth of collateral arteries from preexisting arterioles (arteriogenesis) [2]. Angiogenesis is the process of growth of endothelial sprouts from preexisting postcapillary venules. It is initiated by the vasodilation of venules leading to increased permeability, followed by the proliferation and migration of endothelial cells (ECs). Then, venules are divided by periendothelial cells (intussusception) or are separated by transendothelial cell bridges (bridging) to form capillaries [3, 4].

Arteriogenesis was originally defined as the process of collateral arteries formation which follows the occlusion of a major artery [5]. Arteriogenesis is partly driven by shear stress and involves the proliferation and migration of vascular endothelial cells (ECs) and vascular smooth muscle cells (VSMCs). It leads to the production of mature arteries with a fully developed tunica media, which may provide alternative blood perfusion to ischemic areas [6]. microRNAs (miRNAs) are inhibitory regulators of gene expression which act by binding to complementary messenger RNA (mRNA) transcripts. Following initial studies in developmental biology and cancer, miRNAs have recently come into focus of cardiovascular diagnostics and therapeutics [7, 8]. Since miRNAs repress many target mRNAs, deregulation of one single miRNA can result in a cascade of transcriptional and posttranscriptional changes relevant to disease states [9]. miRNAs have been detected in serum and plasma, and circulating miRNA profiles have now been associated with cancer [10], diabetes [11], and heart disease [12] as an emerging class of biomarkers. Here, we discuss the role of miRNAs in postischemic angiogenesis.

## 2. MicroRNAs Biogenesis

Transcriptions of miRNA genes and protein-coding genes share common regulatory mechanisms [13]. miRNA genes can be embedded in the introns of protein coding genes or can derive from their own transcript units in intergenic regions of the genome. When miRNA genes are located within introns of protein-coding genes, primary miRNA biogenesis is controlled by the same transcriptional mechanisms as the parent gene. In contrast, an independent miRNA gene will have its own transcriptional controls. Interestingly, multiple miRNAs can be produced within a single long primary nuclear miRNA transcript (pri-miRNA transcript), each of which can act independently [14, 15]. The long primary nuclear miRNA transcript (“pri-miRNA”) undergoes maturation by the RNase-III Drosha/Dgcr8 enzyme complex, generating a precursor miRNA (“pre-miRNA”) that is exported from the nucleus by exportin-5 [16]. Cytoplasmic pre-miRNAs are cleaved by a Dicer-containing complex [17] to generate a double-strand miRNA-duplex containing the mature miRNA on one strand and a complementary miRNA* on the other strand. Recently, Dicer-independent miRNA biogenesis pathway has also been reported [18]. The specificity of miRNA targeting is defined by Watson-Crick complementarities between positions 2 to 8 from the 5′ miRNA (also known as the seed sequence), with the 3′ untranslated region (UTR) of their target mRNAs [15]. Besides the binding through 3′ UTR interactions, some miRNAs have been shown to associate to the open reading frame or to the 5′ UTR of the target genes [19]. miRNAs act through formation of stable complexes with proteins of the Argonaute (Ago) family (in particular with Ago2), the core of the RNA-induced silencing complex (RISC). The RISC blocks the translation initiation by competition with the cytoplasmic cap-binding protein eIF4E (eukaryotic translation initiation factor 4E) [20] or the antiassociation factor eIF6, thus preventing the assembly of the ribosome [21]. Besides these mechanisms, RISC-miRNA complexes can move the mRNAs they bind to the “P-bodies,” specialized cytoplasmic compartments enriched in mRNA-catabolizing enzymes, where translational repression or exonucleolytic mRNA degradation or mRNA destabilization may occur [22, 23].

## 3. Expression and Regulation of MicroRNAs in Endothelial Cells

miRNA microarray analysis is, at the moment, the most used and complete technique to analyse global miRNAs expression profile. Using this approach, 28 miRNAs (miRNA-15b, -16, -20, -21, -23a, -23b, -24, -27a, -29a, -30a, -30c, -31, -100, -103, -106, 125a and -b, -126, -181a, -191, -199a, -221, -222, -320, let-7, let-7b, let-7c, and let-7d) have been found to be commonly expressed in ECs cultured under normal conditions [24–30]. The miRNA expression profile in ECs could change if cells are studied under stressed conditions, as we recently proved for miRNA-503 (miR-503), which is induced in ECs in response to diabetes and ischemia [31]. A highly scalable approach using next generation sequencing (small RNA-seq) would facilitate the analysis of miRNA expression patterns in ECs. The group of Martelli was the first to use this approach to analyze the miRNAs expressed by ECs exposed to hypoxia [32]. Based on experimental evidence, it is possible to divide the endothelial miRNAs into pro- and antiangiogenic miRNAs (Table 1).

### 3.1. Proangiogenic MicroRNAs

miR-126 is recognized as the most important miRNA for maintaining vascular integrity during ongoing angiogenesis, as it targets SPRED1 and PIK3R2, two negative regulators of VEGFs signalling [30, 33]. Angiogenic sprouting of vessels requires that the zinc finger transcription factor klf2a induces expression of an endothelial-specific miR-126 [48]. Growth factors increase the expression of the pro-angiogenic miR-130a and miR-296 in ECs [28, 38]. miR-130a stimulates angiogenesis by inhibiting GAX and HOXA5; while, miR-296 acts through the inhibition of hepatocyte growth factor (HGF)-regulated tyrosine kinase [28]. Moreover, EC stimulation with VEGF-A and bFGF lead to a rapid transcription of miR-132. Overexpression of miR-132 increases EC proliferation and *in vitro* networking by targeting p120RasGAP, a GTPase-activating protein [49]. miR-210 overexpression considerably increased HUVEC migration and the formation of capillary-like structures by targeting ephrin A3 [27]. miR-424 promotes angiogenesis by inhibiting cullin 2 (CUL2) and thereby increasing HIF-1*α* levels [36]. The proangiogenesis activity of the miR-17–92 cluster is due to miR-17-5p, miR-18a, and miR-19a [26]. In fact, the latter two miRNAs have been shown to target proteins containing thrombospondin type 1 repeats (TSR) [26, 50]; while miR-17-5p appears to modulate EC migration and proliferation by targeting tissue inhibitor of metalloproteinase 1 (TIMP1) [39]. Recently, the miR-23-27-24 cluster has also been reported to have a prominent role in angiogenesis [40]. In particular, both miR-23 and miR-27 enhance angiogenesis by targeting the anti-angiogenic proteins Sprouty2 and Sema6A [40]. Finally, miR-378 promotes angiogenesis by targeting tumor suppressor candidate 2 (Fus-1) and suppressor of fused (Sufu), thus inducing indirect up-regulation of VEGF and Angiopoietin-1/2 [35].

### 3.2. Antiangiogenic MicroRNAs

The antiangiogenic miR-221/222 was discovered by Poliseno et al. during the first screening of miRNAs in HUVECs [34]. Overexpression of miR-221/222 inhibits the angiogenesis responses to stem cell factor (SCF) by targeting the SCF receptor c-kit [34]. Belonging to miR-17-92 cluster, miR-20a, and miR-92a display antiangiogenenic activity, respectively, targeting VEGF-A and integrin *β*5 (ITGB5) transcripts [44, 45]. Oxidative stress upregulates miR-200c in ECs, which translates in the downmodulation of the miR-200c target ZEB1 [41]. miR-217 and miR-34 regulate the expression of silent information regulator 1 (SirT1). Inhibition of either miR-217 or miR-34 in ECs promotes angiogenesis via an increase in SirT1 activity [42, 43]. Finally, miR-503 expression in ECs is upregulated under anti-angiogenic conditions mimicking diabetes mellitus and ischemia [31]. miR-503 targets cdc25A and cyclinE1 (CCNE1) and its overexpression inhibits EC proliferation, migration, and network formation on Matrigel. Conversely, blocking miR-503 activity restores the angiogenic process in diabetic and ischemic limb muscles. [31].

## 4. MicroRNAs in Postischemic Angiogenesis

**Figure 1:**
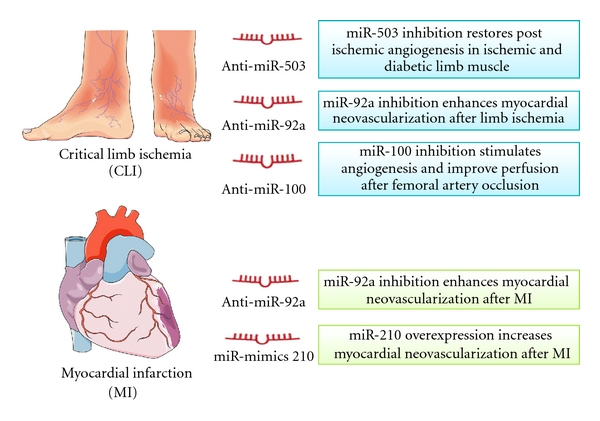
miRNAs-based therapeutic strategies in vascular repairs, There are two major approaches to developing miRNA-based therapeutics: miRNA antagonists and miRNA mimics. miRNA antagonists are generated to inhibit endogenous miRNAs that show a pathogenic gain-of-function in diseased tissues. miRNA mimics are used to upregulate expression of beneficial miRNAs. Inhibition of miR-92a, miR-100 and miR-503 or increase of miR-210 can promote angiogenesis, thus improving postischemic blood flow recovery in limb ischemia or myocardial infarction.

Ischaemic disease is highly prevalent and is associated with elevated morbidity and mortality in developed and developing countries [51]. Functional recovery of ischemic tissues and organs is dependent on establishing collateral networks that supply oxygenated blood [52, 53]. Therapeutic induction of angiogenesis may attenuate ischemic sufferance. Notwithstanding, despite the remarkable results in animal models of limb ischemia and myocardial infarct, the results of 15 years of clinical trials with monotherapeutic proangiogenic agents have been unsatisfactory [54]. One of the reasons accounting for this failure is that monotherapy may be not sufficient to promote and maintain a functional vascular network under severely compromised medical conditions. Thus, one alternative therapeutic approach could consist of drug combinations in order to promote angiogenesis and vascular maturation and stabilization [55]. Because each miRNA has multiple targets, modulating the expression of one miRNA could impact on the complex molecular pathways governing ischemic complications by potentially increasing the expression and/or activity of multiple growth factors. Hence manipulation of miRNAs could represent a novel technology potentially able to interfere with expressional regulation of multiple genes, thus opening new avenues for molecular therapeutics (Figure 1).

### 4.1. Limb Ischemia

Peripheral artery disease (PAD) induces limb muscle hypoperfusion and favours the formation and aggravation of skin ulcers. When the wound healing capacity is lost, PAD patients progress to a critical condition, identified as critical limb ischaemia (CLI), which usually requires minor or major amputations of the affected limb [56]. Despite improvements in surgical treatments, a significant portion of patients with CLI are not eligible for revascularization, and no medical therapy has been shown to be capable of reducing the need for amputation [57]. Diabetes aggravates PAD and CLI. Furthermore, postischemic recovery is delayed in diabetic patients due to diabetes-induced impairment of reparative angiogenesis. A laboratory-based study showed that conditional inactivation of *Dicer* in ECs reduced the spontaneous angiogenic responses to limb ischemia, demonstrating that endothelial miRNAs are required for reparative neovascularisation [26]. Recently, miRNA expression in murine models of limb ischemia has been profiled, showing upregulation of miR-92a and -100 [47, 58]. miR-92 represses the ITGB5 target gene, thus impairing angiogenesis both *in vitro* and *in vivo* [45]. Conversely, miR-100 functions as an endogenous repressor of the serine/threonine protein kinase mammalian target of rapamycin (mTOR), increasing EC proliferation, sprouting activity and network formation on Matrigel [47]. Moreover, we found miR-503 levels to be remarkably higher in ischemic muscles of diabetic mice and of diabetic patients with CLI in comparison to nondiabetic and/or nonischemic controls [31]. In summary, it is evident that a number of miRNAs are modulated by ischemia and participate in postischemic angiogenesis.

### 4.2. Acute Myocardial Infarction

Myocardial ischemic injury results from severe impairment of the coronary blood supply usually produced by thrombosis or other acute alterations of coronary atherosclerotic plaques. Rapid formation of collateral vessels that bypass the obstructed coronary artery is necessary for the survival of the myocardial region that surrounds the necrotic infarct tissue and for the subsequent myocardial repair process.

The most convincing data in the cardiac response to ischemia are for miR-126, miR-210, and miR-92a. Neovascularization at 3 weeks postmyocardial infarct (MI) is inhibited in miR-126 knockout mice in comparison with wild type [33]. Moreover, miR-126 is downregulated in the MI area, but upregulated in the MI border zone [33]. In contrast, miR-210 is upregulated in the ischemic myocardium [46]. miR-210 expression is induced by HIF1*α*, and it has been associated with increased survival of cardiomyocytes and increased angiogenesis in a murine model of myocardial infarction [46]. Finally, miR-92a expression increases after experimental MI and inhibits neovascularisation [45].

### 4.3. miRNAs-Based Therapeutic Strategies in Vascular Repair

There are two major approaches to developing miRNA-based therapeutics: miRNA antagonists and miRNA mimics. miRNA mimics are small, chemically modified, double-stranded RNA molecules that mimic endogenous mature miRNA molecules. Mimics of proangiogenic miRNAs can be used to elevate angiogenesis in the pathological setting of insufficient angiogenesis, such as cardiac and limb ischemia. Caution should be used when overexpressing miRNAs, as an above physiological abundance of a miRNA could result in “off-target effects” through the miRNA binding to seed regions of genes not normally targeted [8]. miRNAs can be overexpressed by using naked oligodeoxynucleotides or viral vectors. Recently, gene therapy using a minicircle DNA vector encoding the miR-210 precursor was shown to enhance myocardial neovascularization in mice with MI [59].

Anti-miRNAs (antimiRs) are modified antisense oligodeoxynucleotides harboring the full or partial complementary reverse sequence of a mature miRNA. These oligodeoxynucleotides are able to reduce the endogenous levels of the miRNA, thus increasing expression of its mRNA targets [60]. Most common chemical modifications to increase antimiR stability are LNA- (locked nucleic acid-) modified nucleotides, in which the 2′-*O*-oxygen is locked into a C3′-endo (RNA) sugar conformation [61] or a complete 2′-*O*-methylation of sugar in a phosphorothioate backbone, and a cholesterol molecule at the 3′ end. These are the so-called “antagomiRs” [62]. LNA-antimiR against miRNAs of the cluster miR-23/24 showed regulation of angiogenesis and choroidal neovascularisation [40]. Systemic delivery of antagomiR was shown to be sufficient to induce efficient and long-lasting miRNA inhibition *in vivo* and to modulate neovascularization [45, 47, 63]. In particular, *in vivo *miR-100 inhibition by specific antagomiRs stimulated angiogenesis and resulted in functional improvement of perfusion after femoral artery occlusion in mice [47]. Similarly, antagomiR-induced inhibition of miR-92a enhanced blood vessel repair in murine models of limb ischemia and MI. [45, 47, 63]. Finally, miRNAs expression could be inhibited using “miRNA sponge” or “decoy.” Typical sponge constructs contain four to ten miRNA binding sites separated by a few nucleotides each to avoid Ago2-mediated endonucleolytic cleavage [64, 65]. By this technique, we inhibited miRNA-503 in the ischemic hindlimb of diabetic mice. This anti-miRNA-based intervention restored postischemic angiogenesis in ischemic and diabetic limb muscles [31].

## 5. Circulating MicroRNAs

The discovery of miRNAs in biological fluids is opening new frontiers in the miRNA arena. Mitchell et al. first demonstrated that circulating miRNAs could be detected reliably in serum and showed that miRNA profiles correlate with specific diseases. Moreover, miRNAs appear protected from RNAse and remain stable in the blood [66]. Therefore, their stability makes miRNAs concentrations well suited for being tested in patient samples as potential biomarkers of different pathological conditions. Recent studies identified miRNAs in different types of microvescicles (MVs) secreted from cultured cells, thus supporting the idea that MVs may serve as physiological carriers of miRNA [11, 67, 68]. In line with this hypothesis, MVs released from cells were proved able to alter gene expression in neighbouring and distant cells *in vitro* and *in vivo* by transferring miRNAs [69, 70]. Two distinct processes of MVs release from the cells have been described. MVs may derive from the endosomal membranes that are extruded from the cell surface of activated cells as exosomes [71]. Furthermore, high-density lipoprotein (HDL) transports endogenous miRNAs and delivers them to recipient cells with functional targeting capabilities [72]. Interestingly, a recent study demonstrated that the majority of circulating miRNAs are complexed with protein rather than with vesicles. In particular, Ago2 was present in human plasma and eluted with plasma miRNAs. This finding raises the possibility that cells release a functional RISC complex into the circulation [73].

Recent reports suggest that serum levels of cardiac miRNA correlate with the severity of heart injury and may represent a good biomarker of MI. Corsten et al. described strikingly increased plasma levels of miR-208b and -499 after MI. However, studies on circulating miRNAs and MI have led to contrasting conclusions on miR-208. Wang et al. suggested miR-208 as a candidate biomarker for MI in humans [74], while Adachi et al. found very low levels of miR-208a and miR-208b in human hearts [75]. The latter result was confirmed by D'Alessandra et al., who reported that miR-208 was detectable only in 30% of MI patients and always at very low levels [76]. In contrast, miR-499 was detectable in all control subjects and increased in all patients with MI [76]. Finally, we found that the plasma levels of miR-503 were dramatically increased in diabetic patients with CLI in comparison with controls. Due to the low number of patients in the study, further analysis is required to investigate miR-503 as a potential circulating biomarker of ongoing ischemia in diabetic subjects [31].

## 6. Future Research

Great interest focuses on exploiting miRNAs for therapeutic purposes and as clinical biomarkers. As therapeutic targets for vascular diseases, miRNAs could represent a major breakthrough because a single miRNA can regulate several target genes, thus influencing multiple molecular pathways. In consideration of the above, miRNAs antagonists or mimics could become an important new class of drugs to regulate angiogenesis. Moreover, manipulation of miRNAs structures might increase their delivery efficiency. In particular, the promising results obtained with modified microparticles or nanoparticles for drug delivery in cancer [37] should be extended to investigate their capacity for promoting vascular regeneration in ischemic tissues. A potential drawback in the therapeutic use of miRNAs is their possible “off-target effects.” Since not all targets of a particular miRNA are disease-related, any therapeutic perturbation of miRNA expression will likely have side effects.

As biomarkers, circulating miRNAs offer a great potential for the diagnosis and prognosis of different diseases. However, there are still limitations in the technology and various issues regarding miRNA measurement and quantification in the circulation. First, circulating miRNA profiling has to be verified in larger and independent studies in order to translate the findings into clinical biomarker. Then, in order to obtain reliable and reproducible results, there is a need to determine suitable normalization methods for blood-based miRNA investigations. Finally, the mechanisms by which miRNAs are released into the circulation and whether circulating miRNA molecules have any functional capabilities need further characterization.

## Figures and Tables

**Table 1:** microRNAs and target genes involved in neovascularisation.

Proangiogenic miRNAs	Target genes	References
miR-126	Spred1/PIK3R2	[33]
miR-221/222	c-kit	[34]
miR-378	Fus-1/Sufu	[35]
miR-424	CUL2	[36]
miR-132	p120RasGAP	[37]
miR-210	EFNA3	[27]
miR-130a	Spred1/PIK3R2	[38]
miR-296	c-kit	[28]
miR-17-5p	Fus-1/Sufu	[39]
miR-23-27	CUL2	[40]

Antiangiogenic miRNAs	Target genes	References

miR-200c	ZEB1	[41]
miR-217	SIRT1	[42]
miR-34	SIRT1	[43]
miR-503	CCNE1/cdc25A	[31]
miR-20a	VEGF-A	[44]
miR-92a	ITGB5	[45]

miRNAs involved in postischemic angiogenesis		

Ischemic stimuli	miRNAs	References

Myocardial infarct	miR-126	[33]
Myocardial infarct Peripheral ischemia	miR-92a	[45]
Myocardial infarct	miR-210	[46].
Peripheral ischemia	miR-503	[31]
Peripheral ischemia	miR-92a	[45]
Myocardial infarct	miR-'100	[47]

## References

[1] Boodhwani M, Munir FW (2009). Therapeutic angiogenesis in diabetes and hypercholesterolemia: influence of oxidative stress. *Antioxidants and Redox Signaling*.

[2] Emanueli C, Madeddu P (2006). Therapeutic angiogenesis: translating experimental concepts to medically relevant goals. *Vascular Pharmacology*.

[3] Adams RH, Alitalo K (2007). Molecular regulation of angiogenesis and lymphangiogenesis. *Nature Reviews Molecular Cell Biology*.

[4] Burri PH, Hlushchuk R, Djonov V (2004). Intussusceptive angiogenesis: its emergence, its characteristics, and its significance. *Developmental Dynamics*.

[5] Scholz D, Cai WJ, Schaper W (2001). Arteriogenesis, a new concept of vascular adaptation in occlusive disease. *Angiogenesis*.

[6] Smart N, Dubé KN, Riley PR (2009). Coronary vessel development and insight towards neovascular therapy. *International Journal of Experimental Pathology*.

[7] Small EM, Olson EN (2011). Pervasive roles of microRNAs in cardiovascular biology. *Nature*.

[8] Van Rooij E (2011). The art of MicroRNA research. *Circulation Research*.

[9] Inui M, Martello G, Piccolo S (2010). MicroRNA control of signal transduction. *Nature Reviews Molecular Cell Biology*.

[10] Reid G, Kirschner MB, van Zandwijk N (2011). Circulating microRNAs: association with disease and potential use as biomarkers. *Critical Reviews in Oncology/Hematology*.

[11] Zampetaki A, Kiechl S, Drozdov I (2010). Plasma MicroRNA profiling reveals loss of endothelial MiR-126 and other MicroRNAs in type 2 diabetes. *Circulation Research*.

[12] Gupta SK, Bang C, Thum T (2010). Circulating MicroRNAs as biomarkers and potential paracrine mediators of cardiovascular disease. *Circulation*.

[13] Davis BN, Hata A (2009). Regulation of microRNA biogenesis: a miRiad of mechanisms. *Cell Communication and Signaling*.

[14] Zeng Y, Cullen BR (2006). Recognition and cleavage of primary microRNA transcripts. *Methods in Molecular Biology*.

[15] Bartel DP (2004). MicroRNAs: genomics, biogenesis, mechanism, and function. *Cell*.

[16] Yi R, Qin Y, Macara IG, Cullen BR (2003). Exportin-5 mediates the nuclear export of pre-microRNAs and short hairpin RNAs. *Genes and Development*.

[17] Hutvágner G, McLachlan J, Pasquinelli AE, Bálint E, Tuschl T, Zamore PD (2001). A cellular function for the RNA-interference enzyme dicer in the maturation of the let-7 small temporal RNA. *Science*.

[18] Cifuentes D, Xue H, Taylor DW (2010). A novel miRNA processing pathway independent of dicer requires argonaute2 catalytic activity. *Science*.

[19] Ørom UA, Nielsen FC, Lund AH (2008). MicroRNA-10a binds the 5′UTR of ribosomal protein mRNAs and enhances their translation. *Molecular Cell*.

[20] Kiriakidou M, Tan GS, Lamprinaki S, De Planell-Saguer M, Nelson PT, Mourelatos Z (2007). An mRNA m7G cap binding-like motif within human Ago2 represses translation. *Cell*.

[21] Chendrimada TP, Finn KJ, Ji X (2007). MicroRNA silencing through RISC recruitment of eIF6. *Nature*.

[22] Eulalio A, Behm-Ansmant I, Izaurralde E (2007). P bodies: at the crossroads of post-transcriptional pathways. *Nature Reviews Molecular Cell Biology*.

[23] Parker R, Sheth U (2007). P bodies and the control of mRNA translation and degradation. *Molecular Cell*.

[24] Kuehbacher A, Urbich C, Zeiher AM, Dimmeler S (2007). Role of Dicer and Drosha for endothelial microRNA expression and angiogenesis. *Circulation Research*.

[25] Suárez Y, Fernández-Hernando C, Pober JS, Sessa WC (2007). Dicer dependent microRNAs regulate gene expression and functions in human endothelial cells. *Circulation Research*.

[26] Suárez Y, Fernández-Hernando C, Yu J (2008). Dicer-dependent endothelial microRNAs are necessary for postnatal angiogenesis. *Proceedings of the National Academy of Sciences of the United States of America*.

[27] Fasanaro P, D’Alessandra Y, Di Stefano V (2008). MicroRNA-210 modulates endothelial cell response to hypoxia and inhibits the receptor tyrosine kinase ligand ephrin-A3. *Journal of Biological Chemistry*.

[28] Würdinger T, Tannous BA, Saydam O (2008). miR-296 regulates growth factor receptor overexpression in angiogenic endothelial cells. *Cancer Cell*.

[29] Chen C, Chai H, Wang X (2008). Soluble CD40 ligand induces endothelial dysfunction in human and porcine coronary artery endothelial cells. *Blood*.

[30] Fish JE, Santoro MM, Morton SU (2008). miR-126 regulates angiogenic signaling and vascular integrity. *Developmental Cell*.

[31] Caporali A, Meloni M, Völlenkle C (2011). Deregulation of microRNA-503 contributes to diabetes mellitus-induced impairment of endothelial function and reparative angiogenesis after Limb Ischemia. *Circulation*.

[32] Voellenkle C, van Rooij J, Guffanti A (2012). Deep-sequencing of endothelial cells exposed to hypoxia reveals the complexity of known and novel microRNAs. *RNA*.

[33] Wang S, Aurora AB, Johnson BA (2008). The endothelial-specific microRNA miR-126 governs vascular integrity and angiogenesis. *Developmental Cell*.

[34] Poliseno L, Tuccoli A, Mariani L (2006). MicroRNAs modulate the angiogenic properties of HUVECs. *Blood*.

[35] Lee DY, Deng Z, Wang CH, Yang BB (2007). MicroRNA-378 promotes cell survival, tumor growth, and angiogenesis by targeting SuFu and Fus-1 expression. *Proceedings of the National Academy of Sciences of the United States of America*.

[36] Ghosh G, Subramanian IV, Adhikari N (2010). Hypoxia-induced microRNA-424 expression in human endothelial cells regulates HIF-*α* isoforms and promotes angiogenesis. *Journal of Clinical Investigation*.

[37] Anand S, Majeti BK, Acevedo LM (2010). MicroRNA-132-mediated loss of p120RasGAP activates the endothelium to facilitate pathological angiogenesis. *Nature Medicine*.

[38] Chen Y, Gorski DH (2008). Regulation of angiogenesis through a microRNA (miR-130a) that down-regulates antiangiogenic homeobox genes GAX and HOXA5. *Blood*.

[39] Otsuka M, Zheng M, Hayashi M (2008). Impaired microRNA processing causes corpus luteum insufficiency and infertility in mice. *Journal of Clinical Investigation*.

[40] Zhou Q, Gallagher R, Ufret-Vincenty R, Li X, Olson EN, Wang S (2011). Regulation of angiogenesis and choroidal neovascularization by members of microRNA-23*∼*27*∼*24 clusters. *Proceedings of the National Academy of Sciences of the United States of America*.

[41] Magenta A, Cencioni C, Fasanaro P (2011). MiR-200c is upregulated by oxidative stress and induces endothelial cell apoptosis and senescence via ZEB1 inhibition. *Cell Death and Differentiation*.

[42] Menghini R, Casagrande V, Cardellini M (2009). MicroRNA 217 modulates endothelial cell senescence via silent information regulator 1. *Circulation*.

[43] Zhao T, Li J, Chen AF (2010). MicroRNA-34a induces endothelial progenitor cell senescence and impedes its angiogenesis via suppressing silent information regulator 1. *American Journal of Physiology*.

[44] Hua Z, Lv Q, Ye W (2006). Mirna-directed regulation of VEGF and other angiogenic under hypoxia. *PLoS ONE*.

[45] Bonauer A, Carmona G, Iwasaki M (2009). MicroRNA-92a controls angiogenesis and functional recovery of ischemic tissues in Mice. *Science*.

[46] Hu S, Huang M, Li Z (2010). MicroRNA-210 as a novel therapy for treatment of ischemic heart disease. *Circulation*.

[47] Grundmann S, Hans FP, Kinniry S (2011). MicroRNA-100 regulates neovascularization by suppression of mammalian target of rapamycin in endothelial and vascular smooth muscle cells. *Circulation*.

[48] Nicoli S, Standley C, Walker P, Hurlstone A, Fogarty KE, Lawson ND (2010). MicroRNA-mediated integration of haemodynamics and Vegf signalling during angiogenesis. *Nature*.

[49] Anand S, Majeti BK, Acevedo LM (2010). MicroRNA-132-mediated loss of p120RasGAP activates the endothelium to facilitate pathological angiogenesis. *Nature Medicine*.

[50] Dews M, Homayouni A, Yu D (2006). Augmentation of tumor angiogenesis by a Myc-activated microRNA cluster. *Nature Genetics*.

[51] Selvin E, Erlinger TP (2004). Prevalence of and risk factors for peripheral arterial disease in the United States: results from the National Health and Nutrition Examination Survey, 1999-2000. *Circulation*.

[52] Takeshita S, Pu LQ, Stein LA (1994). Intramuscular administration of vascular endothelial growth factor induces dose-dependent collateral artery augmentation in a rabbit model of chronic limb ischemia. *Circulation*.

[53] Emanueli C, Salis MB, Pinna A, Graiani G, Manni L, Madeddu P (2002). Nerve growth factor promotes angiogenesis and arteriogenesis in ischemic hindlimbs. *Circulation*.

[54] Henry TD, Annex BH, McKendall GR (2003). The VIVA trial: vascular endothelial growth factor in ischemia for vascular angiogenesis. *Circulation*.

[55] Emanueli C, Madeddu P (2005). Changing the logic of therapeutic angiogenesis for ischemic disease. *Trends in Molecular Medicine*.

[56] Slovut DP, Sullivan TM (2008). Critical limb ischemia: medical and surgical management. *Vascular Medicine*.

[57] Norgren L, Hiatt WR, Dormandy JA (2007). Inter-Society Consensus for the management of peripheral arterial disease (TASC II). *International Angiology*.

[58] Greco S, De Simone M, Colussi C (2009). Common micro-RNA signature in skeletal muscle damage and regeneration induced by Duchenne muscular dystrophy and acute ischemia. *FASEB Journal*.

[59] Hu S, Huang M, Li Z (2010). MicroRNA-210 as a novel therapy for treatment of ischemic heart disease. *Circulation*.

[60] Stenvang J, Kauppinen S (2008). MicroRNAs as targets for antisense-based therapeutics. *Expert Opinion on Biological Therapy*.

[61] Prakash TP, Bhat B (2007). 2′-modified oligonucleotides for antisense therapeutics. *Current Topics in Medicinal Chemistry*.

[62] Krützfeldt J, Rajewsky N, Braich R (2005). Silencing of microRNAs in vivo with “antagomirs”. *Nature*.

[63] Van Solingen C, Seghers L, Bijkerk R (2009). Antagomir-mediated silencing of endothelial cell specific microRNA-126 impairs ischemia-induced angiogenesis. *Journal of Cellular and Molecular Medicine*.

[64] Gentner B, Schira G, Giustacchini A (2009). Stable knockdown of microRNA in vivo by lentiviral vectors. *Nature Methods*.

[65] Ebert MS, Neilson JR, Sharp PA (2007). MicroRNA sponges: competitive inhibitors of small RNAs in mammalian cells. *Nature Methods*.

[66] Mitchell PS, Parkin RK, Kroh EM (2008). Circulating microRNAs as stable blood-based markers for cancer detection. *Proceedings of the National Academy of Sciences of the United States of America*.

[67] Hunter MP, Ismail N, Zhang X (2008). Detection of microRNA expression in human peripheral blood microvesicles. *PLoS ONE*.

[68] Collino F, Deregibus MC, Bruno S (2010). Microvesicles derived from adult human bone marrow and tissue specific mesenchymal stem cells shuttle selected pattern of miRNAs. *PLoS ONE*.

[69] Zhang Y, Liu D, Chen X (2010). Secreted monocytic miR-150 enhances targeted endothelial cell migration. *Molecular Cell*.

[70] Zernecke A, Bidzhekov K, Noels H (2009). Delivery of microRNA-126 by apoptotic bodies induces CXCL12-dependent vascular protection. *Science Signaling*.

[71] Théry C, Zitvogel L, Amigorena S (2002). Exosomes: composition, biogenesis and function. *Nature Reviews Immunology*.

[72] Vickers KC, Palmisano BT, Shoucri BM, Shamburek RD, Remaley AT (2011). MicroRNAs are transported in plasma and delivered to recipient cells by high-density lipoproteins. *Nature Cell Biology*.

[73] Arroyo JD, Chevillet JR, Kroh EM (2011). Argonaute2 complexes carry a population of circulating microRNAs independent of vesicles in human plasma. *Proceedings of the National Academy of Sciences of the United States of America*.

[74] Wang GK, Zhu JQ, Zhang JT (2010). Circulating microRNA: a novel potential biomarker for early diagnosis of acute myocardial infarction in humans. *European Heart Journal*.

[75] Adachi T, Nakanishi M, Otsuka Y (2010). Plasma microRNA 499 as a biomarker of acute myocardial infarction. *Clinical Chemistry*.

[76] D’Alessandra Y, Devanna P, Limana F (2010). Circulating microRNAs are new and sensitive biomarkers of myocardial infarction. *European Heart Journal*.

